# Multicenter ^18^F-PI-2620 PET for In Vivo Braak Staging of Tau Pathology in Alzheimer’s Disease

**DOI:** 10.3390/biom12030458

**Published:** 2022-03-16

**Authors:** Michael Rullmann, Matthias Brendel, Matthias L. Schroeter, Dorothee Saur, Johannes Levin, Robert G. Perneczky, Solveig Tiepolt, Marianne Patt, Andre Mueller, Victor L. Villemagne, Joseph Classen, Andrew W. Stephens, Osama Sabri, Henryk Barthel, on behalf of the German Imaging Initiative for Tauopathies (GII4T)

**Affiliations:** 1 Department of Nuclear Medicine, University of Leipzig, 04103 Leipzig, Germany; solveig.tiepolt@medizin.uni-leipzig.de (S.T.); marianne.patt@medizin.uni-leipzig.de (M.P.); osama.sabri@medizin.uni-leipzig.de (O.S.); henryk.barthel@medizin.uni-leipzig.de (H.B.); 2 Department of Nuclear Medicine, University Hospital of Munich, LMU Munich, 80336 Munich, Germany; matthias.brendel@med.uni-muenchen.de; 3 Clinic for Cognitive Neurology, University of Leipzig, 04103 Leipzig, Germany; schroet@cbs.mpg.de; 4 Max Planck Institute for Human Cognitive and Brain Sciences, 04103 Leipzig, Germany; 5 Department of Neurology, University of Leipzig, 04103 Leipzig, Germany; dorothee.saur@medizin.uni-leipzig.de (D.S.); joseph.classen@medizin.uni-leipzig.de (J.C.); 6 Department of Neurology, University Hospital of Munich, LMU Munich, 81377 Munich, Germany; johannes.levin@med.uni-muenchen.de; 7 Munich Cluster for Systems Neurology (SyNergy), 81377 Munich, Germany; robert.perneczky@med.uni-muenchen.de; 8 German Center for Neurodegenerative Disorders (DZNE) Munich, 81377 Munich, Germany; 9 Department of Psychiatry and Psychotherapy, University Hospital, LMU Munich, 80336 Munich, Germany; 10 Ageing Epidemiology (AGE) Research Unit, School of Public Health, Imperial College London, London W6 8RP, UK; 11 Sheffield Institute for Translational Neuroscience (SITraN), University of Sheffield, Sheffield S10 2HQ, UK; 12 Life Molecular Imaging GmbH, 13353 Berlin, Germany; a.mueller@life-mi.com (A.M.); a.stephens@life-mi.com (A.W.S.); 13 Department of Psychiatry, University of Pittsburgh, Pittsburgh, PA 15213, USA; victor.villemagne@pitt.edu

**Keywords:** Alzheimer’s disease, Tau, PET, Braak staging, PI-2620

## Abstract

Tau aggregates accumulate in the Alzheimer’s disease (AD) brain according to the established Braak staging scheme and spread from transentorhinal over limbic regions to the neocortex. To impact the management of AD patients, an in vivo tool for tau Braak staging is needed. First-generation tau tracers have limited performance in detecting early stages of tau. Therefore, we tested the corresponding capability of the next-generation tau tracer, ^18^F-PI-2620. We analyzed ^18^F-PI-2620 multicenter PET data from 37 beta-amyloid-positive AD dementia patients and those from 26 healthy controls. We applied kinetic modeling of the 0–60 min p.i. PET data using MRTM2 with the lower cerebellum as the reference region to extract Braak stage-dependent distribution volume ratios, whereas controls were used to define Braak stage PET positivity thresholds. Stage-dependent PET positivity widely followed the Braak scheme (except Braak stage III) presenting descending frequency of PET positivity from Braak I (43%), II (38%), III (49%), IV (35%), V (30%) to VI (14%). A strictly hierarchical model was met by 64% of AD dementia cases. Nineteen percent showed a hippocampal sparing tauopathy pattern. Thus, we could assign 87% to the six-stage hierarchical Braak model including tauopathy variants. ^18^F-PI-2620 PET appears to be able to perform Braak tau staging of AD in vivo.

## 1. Introduction

Tau neurofibrillary tangles accumulate in Alzheimer’s disease (AD) brain in a stereo-typical pattern according to the established Braak staging scheme and spread from trans-entorhinal cortex over entorhinal and hippocampal cortices medially, then laterally around temporal lobe and finally into association and primary sensory regions of the frontal, parietal and occipital cortices [1,2,3,4]. Since Braak staging is also closely associated with cognitive impairment in AD, the staging scheme is part of the NIA-AA guidelines [5]. Braak staging is used to classify patients suffering from AD according to their propagation and progression of tau pathology, which ideally supports AD management and clinical trials as a framework to characterize the individual disease stage.

In vivo studies using early-generation tau tracers allowed for the first time to visualize anatomical distribution of tau directly in the living human brain [6,7]. Unfortunately, off-target binding, a key problem of those first-generation tau tracers, restrained the performance in detecting early tau stages [8,9,10,11]. Second-generation tau tracer overcome those drawbacks [12,13] and should be tested if they can recapitulate early and late Braak stages in vivo.

^18^F-PI-2620 is a next-generation tau imaging tracer with high affinity to 3/4R tau in AD [13], showing significant results in discrimination of patients with AD [14], excellent selectivity and proven absent off-target binding [13]. In the present study, we aimed to assess the capability of detecting early and late Braak stages with the second-generation tau PET tracer ^18^F-PI-2620 in vivo. We hypothesized that ^18^F-PI-2620 PET can detect tau accumulations in each single Braak stage in a descending distribution of the stages, so that we can map the vast majority of cases into the Braak scheme. We also expected an association of Braak staging and its respective tau accumulation to global cognition. 

## 2. Materials and Methods

We included 38 patients with probable AD dementia according to the NINCDS–ADRDA criteria and 26 healthy controls (HC) in the analysis of this study. All patients with AD dementia showed amyloid pathophysiology as indicated by low beta-amyloid in CSF or amyloid deposition in PET. All participants (or their legal representatives) provided written informed consent. We recruited all subjects at four different centers in three different countries (Leipzig, Germany, AD *n* = 14; Melbourne, Australia, HC *n* = 5; Munich, Germany, AD *n* = 12, HC *n* = 11; New Haven, USA, AD *n* = 12, HC *n* = 10). The study was conducted according to the guidelines of the Declaration of Helsinki, and was approved by the Institutional Review Board (or Ethics Committee) of LMU Munich (application numbers 17-569 and 19-022). 

Radiosynthesis of ^18^F-PI-2620 was done as described elsewhere [15]. 

PET imaging with ^18^F-PI-2620 was collected on different scanners with established standard parameters at the above mentioned specialized PET imaging sites as previously described [15]. Briefly, after intravenous injection of a single dose of ~300 MBq ^18^F-PI-2620, dynamic brain imaging was performed (0–60 min p.i.). Resulting dynamic PET data were reconstructed into 23 frames: 6 × 30 s, 4 × 60 s, 4 × 120 s and 9 × 300 s. All dynamic images were corrected for motion and registered to their individual T1-MRI map, if available. To generate parametric images expressed as distribution volume ratio (DVR) we applied MRTM2 [16] with lower cerebellum as reference region using PMOD (version 3.5, PMOD Technologies LLC, Zurich, Switzerland). For atlas application, we calculated the spatial normalization and its inverse transformation, which we used to transform the atlas file to the individual space. Spatial normalization was either computed MR-based or, if no individual MRI map was available, based on an averaged early PET map (sum of the first 15 min p.i.). 

We applied seven regions of interest [8], which closely correspond to the staging scheme defined by Braak and Braak [1]. In vivo Braak stages were categorized according to the decision rules of Schwarz et al. [8]: Transentorhinal cortex (Braak stage I), Hippocampus (Braak stage II), fusiform gyrus (Braak stage III), middle temporal gyrus and extrastriate visual cortex (Braal stage IV), superior temporal gyrus (Braak stage V), primary visual cortex (Braak stage VI). Positivity for each Braak stage was defined as DVRs above mean value plus 2.5 times the standard deviation of the healthy controls, which corresponds to a theoretical maximum of ≈1.2% false positive. Additionally, we condensed the six-stage model data into an established three-class scheme (B1 for Braak stage I + II, B2 for III + IV and B3 for V + VI) [5]. We also implemented a hierarchical scheme, where a stage may only be positive if the previous stage was also positive. Other tauopathy variants, like hippocampal and visual cortex sparing, were defined inside the hierarchical rules and allowed a negative Braak stage I or II, and V or VI, respectively.

Application of Braak atlas and statistics was done in Matlab (version 9.6, The Mathworks Inc., Natick, MA, USA) and SPM (version 12, The Wellcome Centre for Human Neuroimaging, University College London, UK) if applicable. Demographic parameters were compared using Student’s *t* test or exact Fisher test. Associations between Braak stage and global measure of cognition (MMSE) were assessed with either Pearson’s correlation or partial correlations to correct for confounding effects of age. We also computed sensitivity for discriminating AD dementia cases in three scenarios: based on B1–B3 positivity, B2–B3 positivity and B3 positivity.

## 3. Results

In both of our examined groups, we found no sex or age differences, whereas MMSE was significantly lower in the AD dementia group (Table 1).

**Figure 1:**
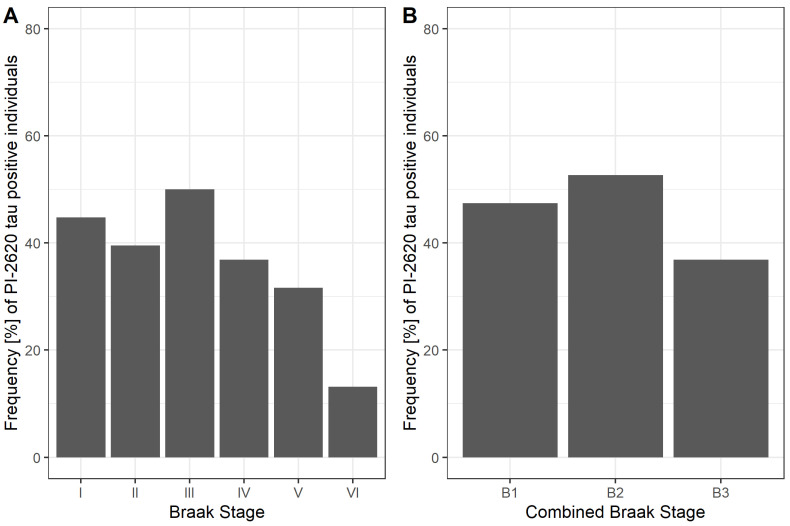
Frequency of ^18^F-PI-2620 tau PET positivity for (**A**) each Braak stage and (**B**) the combined Braak stage model.

**Figure 2:**
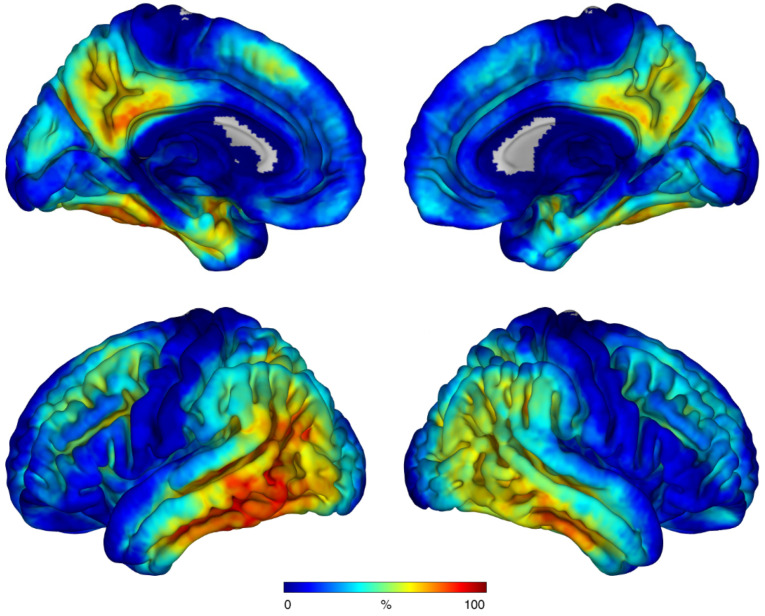
Voxel-wise frequency map of tau positivity of all voxels from all AD dementia cases projected on a cortex surface.

**Figure 3:**
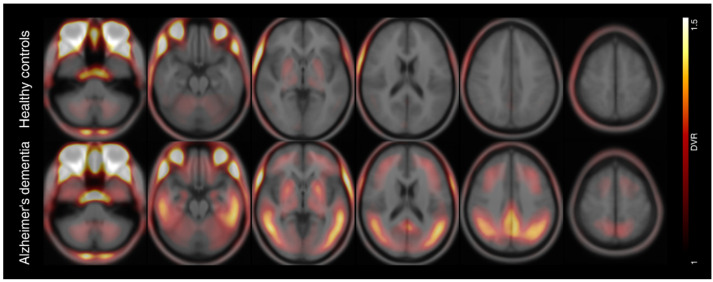
Mean parametric ^18^F-PI-2620 PET distribution volume ratio (DVR) images of healthy controls (top row) and patients with Alzheimer’s dementia (bottom row) overlayed on an averaged anatomical MRI scan in standard space of the Montreal Neurological Institute (MNI).

Braak stage-dependent positivity was defined at following DVR thresholds: 1.29, 1.13, 1.21, 1.24, 1.23 and 1.18. Except for Braak stage III, PET positivity widely followed the Braak scheme (Figure 1A) and its frequency declined from Braak I (45%), II (39%), III (50%), IV (37%), V (32%) to VI (13%). The combined three-class scheme showed similar behavior (Figure 1B). Both frequency distributions underpinned that ^18^F-PI-2620 depositions begins in Braak stage I with its final stage at Braak VI. A voxel-wise frequency mapping representing the amount of tau positive voxels in the whole brain confirmed that distribution (Figure 2). Mean parametric DVR images of both cohorts are shown in Figure 3.

Transferring the data into a hierarchical model, 65% of the AD dementia cases met the hierarchical criterion in the six-stage and 79% in the three-class model. 

**Figure 4:**
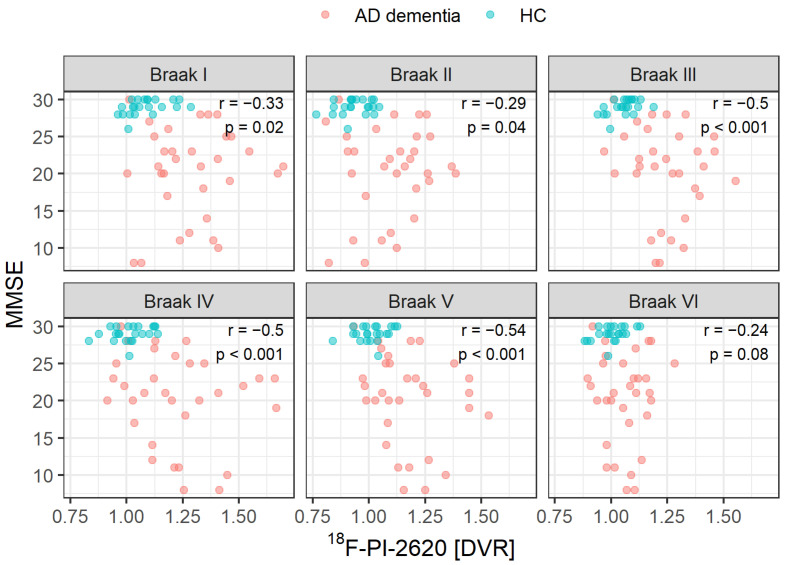
Association between ^18^F-PI-2620 DVR and MMSE score. Partial correlation was adjusted for age. AD dementia: participants with Alzheimer’s dementia. HC: healthy controls.

We found no correlation of highest Braak stage with either age (R = −0.28, *p* = 0.09) or MMSE (R = 0.24, *p* = 0.2), but within each stage we found negative correlations of DVR and MMSE while correcting for age except for Braak VI (Figure 4).

The hippocampal sparing tauopathy variant was found in seven (18%) and six (16%) cases for the six- and three-class scheme, respectively. Sole visual cortex sparing was not evident in our cohort. A combination of both tauopathy variants was present in one case (3%).

All in all, we could assign 33 AD dementia cases (87%) to the six-stage and 36 AD dementia cases (95%) to the three-stage hierarchical Braak model including tauopathy variants.

**Figure 5:**
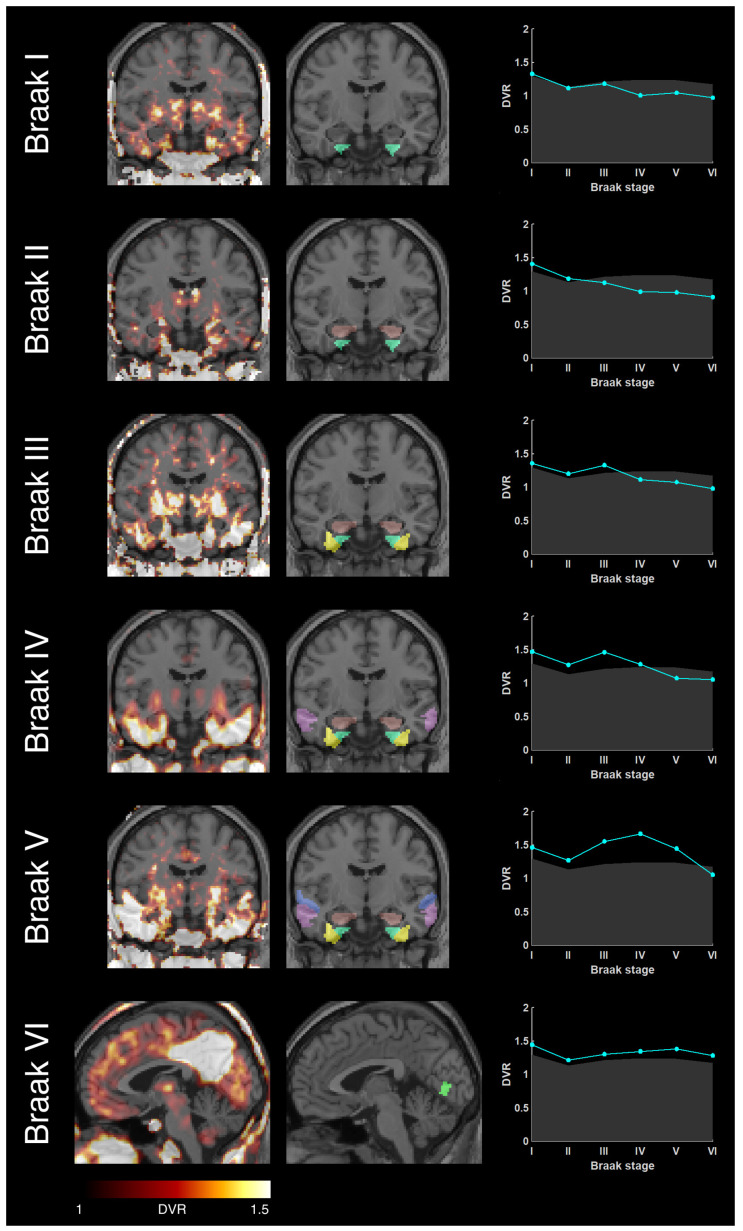
Example AD dementia cases for each Braak stage showing parametric tau PET image fused with a standard MRI (left), MRI including Braak atlas regions (middle) and individual Braak profile (right). The plot shows mean DVR values of each Braak stage (cyan) in relation to the normal DVR (gray) defined by the healthy controls.

Example cases for each Braak stage are shown in Figure 5. For each stage, an example fused PET-MR image is presented along with the corresponding Braak atlas region and the individual Braak profile plot.

Sensitivity of ^18^F-PI-2620 tau PET for discriminating AD dementia patients from healthy controls increased from 37% when considering positivity in the B3 stage as positivity for AD dementia to 58% when including the B2 stage, and to 66% when including the B1+B2 stages, with the specificity values equaling 96%.

## 4. Discussion

We assessed whether the next-generation PET tracer ^18^F-PI-2620 is able to detect the histopathologically defined Braak stages in vivo in a cohort of 38 amyloid-positive AD dementia patients. We found that ^18^F-PI-2620 accumulation widely recapitulates the six-stage Braak model [1,2,3,4] including variants of visual cortex and hippocampal sparing [17].

The overwhelming majority of our cases categorized by its ^18^F-PI-2620 tau depositions followed the histological patterns proposed Braak [1]. Those findings are in line with previous studies using ^18^F-flortaucipir [8] or ^18^F-MK-6240 [12]. In comparison to the latter study, we only included beta-amyloid positive participants, which is why we cannot compare their findings regarding tau accumulation in absence of prominent beta-amyloid pathology. Pascoal et al. [12] emphasize the application of a multi-stage in vivo system (e.g., Braak staging) in favor of a dichotomization into tau positive and negative classes as used in the FDA package insert of ^18^F-flortaucipir [18]. This would allow therapeutic effects to be monitored more precisely in clinical trials or provide a more detailed characterization of longitudinal tau progression [19]. The underlying topographical neurofibrillary tangle progression in AD is suggested to be supported trans-synaptically by misfolded tau protein [20] and trans-neurally by network connections [21]. Recent findings relate the tau propagation to microglial activation, which jointly spread along the Braak stages [22]. In comparison to ^18^F-flortaucipir and their dichotomization approach [18], where a positive scan was defined as a B3 level of tau pathology, we also demonstrated that additional inclusion of B1 and B2 levels increased sensitivity for distinguishing AD dementia.

The Braak staging scheme represents certain steps of disease progression but does not incorporate DVR intensity variations above the binarization threshold, whereas an elevated DVR signal could offer relevant extra information. E.g., MMSE values correlated with DVR values but not with the corresponding highest Braak stage, indicating that the DVR tau signal reveals more information than the pure level of tau spreading in the brain itself.

A similar quantization aspect is imaginable on a histopathological level. While the applied Braak atlas had the goal of replicating Braak’s histopathological sections as closely as possible [8], the potential of whole brain in vivo quantification had not been fully exploited. Instead of a rather limited selection of sections, imaging studies could assess a broader spectrum of regions that can be fully quantified to reveal additional information about intra-individual spreading.

We found a relation of DVR signal intensity and MMSE, a measure of global cognition in our cohort of amyloid positive participants with Alzheimer’s dementia. Here, higher DVR tau signal was associated with worse MMSE scores in each Braak stage except stage VI, where we only found a statistical trend. This finding is consistent with the hypothesis that tau is a significant impetus of the AD progression [4,23,24] and is in line with the known floor effects of the MMSE in advanced disease stages. Since all of our AD dementia patients were beta-amyloid positive (A+ according to [25]), we did not observe cases of primary age-related tauopathy (PART) [26] or suspected non-alzheimer’s pathology (SNAP) [27]. Compared to MMSE, more specific measurement tools for cognition could deliver a more detailed view on the relation of Braak level progressing, tau load and specific cognitive functions. It also seems worth studying tau Braak staging not only in classical AD but also in patients with MCI or AD subtypes such as visual-variants (PCA), language-variant (LPA) or genetic variants (ADAD) to distinguish potential subtype profiles as already suggested [28].

We found no significant age effect on highest Braak stage in our cohort. While other groups found divergent effects of age related tau signal, our sample size is substantially smaller compared to e.g., Lowe et al. [28], who found modest correlations: positive in cognitively unimpaired (*n* = 601), negative in cognitively impaired (*n* = 86). Also other groups report age effects at least in the medial temporal lobe [29,30], but there are also studies, which found no age association with tau PET signal [8,31]. In all those studies, the proposed relations seem to be coupled to beta-amyloid status and cognitive impairment. A comparison to our demented beta-amyloid positive AD participants is complex, but in line with Lowe et al. [28] and Pontecorvo et al. [32], if we interpret our negative correlation with *p* = 0.09 as a statistical trend being aware of our smaller sample size.

Some limitations of this present study should be mentioned. We applied an atlas-based approach to assess DVR signal in each Braak stage. While transforming the atlas template to the individual brain, minor discrepancies are possible after non-linear transformation. A gray matter masking, which could improve quantification, was not considered since an MRI dataset was not available for all participants. The atlas itself was exactly applied as introduced by Schwarz and colleagues [8]. While an atlas optimization was outside the scope of this study, it is conceivable, that some ROIs are potentially biased by off-target binding (e.g., Braak III by choroid plexus) and an optimized data-driven ROI staging approach could improve Braak staging. Instead of SUVR maps, we used kinetic modeling and its resulting DVR maps for Braak staging. While we do not see this as a limitation, we do recognize the problem of applying kinetic modeling in clinical practice and thus the limited transferability of our results to the clinic. In our multicenter study, different PET scanners with different reconstruction methods were used to acquire ^18^F-PI-2620 data. We did not evaluate or correct for that multicenter effect, but rather see this as a strength of our study and demonstrate the applicability of in vivo Braak staging independent of technical setup. The MMSE was the only psychometric endpoint available across the included cohorts, restricting more detailed cognitive phenotyping due to the limited sensitivity, especially in early disease stages. At the same time, the MMSE was shown to correlate well with the dementia stages of AD [33].

To conclude, ^18^F-PI-2620 tau PET seems capable of Braak tau staging in AD in vivo providing a marker of tau deposition and disease progress. It seems worth expanding the concept of in vivo tau staging by ^18^F-PI-2620 to other tauopathies apart from AD.

## Figures and Tables

**Table 1:** Demographic information of all participants.

Parameter	Patients with Alzheimer’s Dementia	Healthy Controls	*p*
N	38	26	
Sex (female/male)	21/17	13/13	0.8 ^1^
Age (years)	69 ± 9	67 ± 11	0.24 ^2^
MMSE (if available)	20 ± 6 (*n* = 32)	29 ± 1 (*n* = 22)	6 × 10^−8 2^

^1^ Exact Fisher test. ^2^ Student’s *t* test.

## Data Availability

The data presented in this study are available on request from the corresponding author. The data are not publicly available due to their containing information that could compromise the privacy of the participants.
